# Supplementary material for: Blood Metabolic Biomarkers of Diabetes Mellitus Type 2 in Aged Adults Determined by a UPLC-MS Metabolomic Approach
Source: Metabolites. 2025 Jun 12;15(6):395. doi: 10.3390/metabo15060395 (PMC12195025; doi:10.3390/metabo15060395)
Supplement: Supplementary file 1 [file metabolites-15-00395-s001.zip › Supplementary_Figure_S1.pdf]

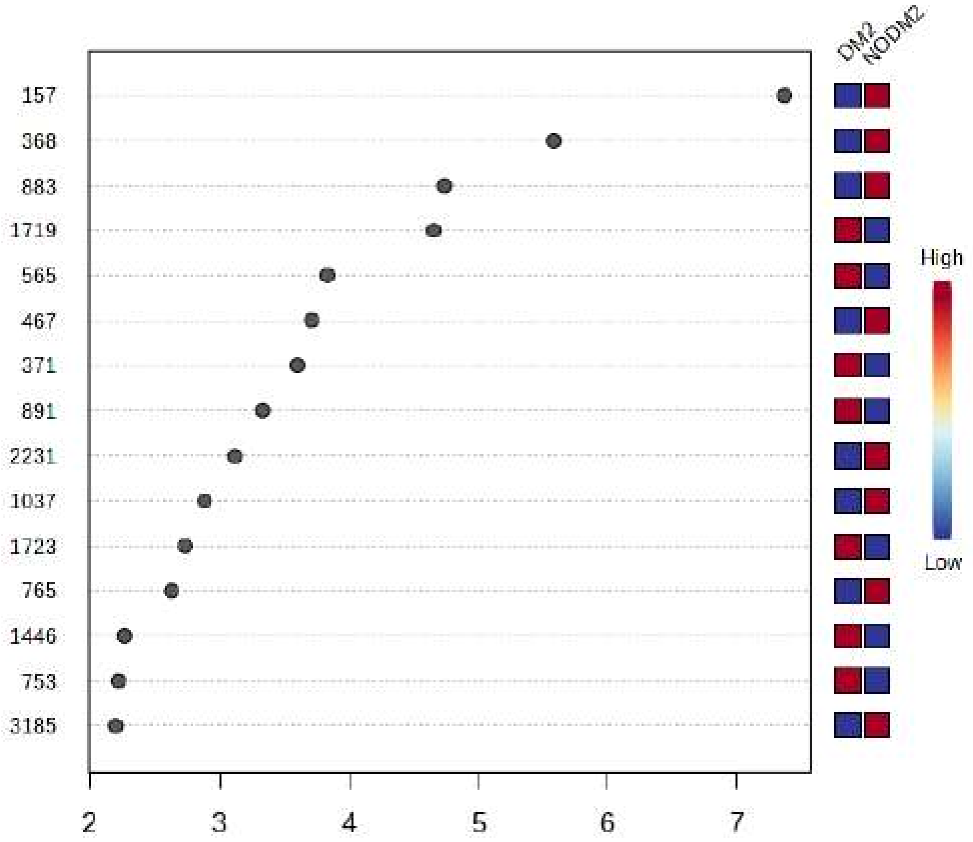

Figure S1. Important features as obtained from the PLS-DA done with MetaboAnalyst.

Left axes codes correspond to the features (retention time_m/z) or identified metabolite as follows (see EXCEL file): 157, LPC(16:0); 368, LPC(18:2); 883, 9.85_583.25; 1719, PC(16:0/18:2); 565, LPC(20:4); 467, 3.43_531.82 (unidentified 1); 371, 3.90_520.83; 891, 9.87_584.26; 2231, 12.40_828.55; 1037, Glycine-Histidine; 1723, PC(16:0/18:2); 765, 7.86_570.43; 1446, 10.28_229.14; 753, LPC(22:6); 3185, LPC(14:0).
