# Supplementary material for: Blood Metabolic Biomarkers of Diabetes Mellitus Type 2 in Aged Adults Determined by a UPLC-MS Metabolomic Approach
Source: Metabolites. 2025 Jun 12;15(6):395. doi: 10.3390/metabo15060395 (PMC12195025; doi:10.3390/metabo15060395)

Supplementary Figure S2. Score-plots of the UPLC-MS data obtained after processing with partial least square discriminant analysis (PLS-DA) using the softwares XS application (upper panel) and MetaboAnalyst (lower panel). Sample 47 was considered outlier and excluded.

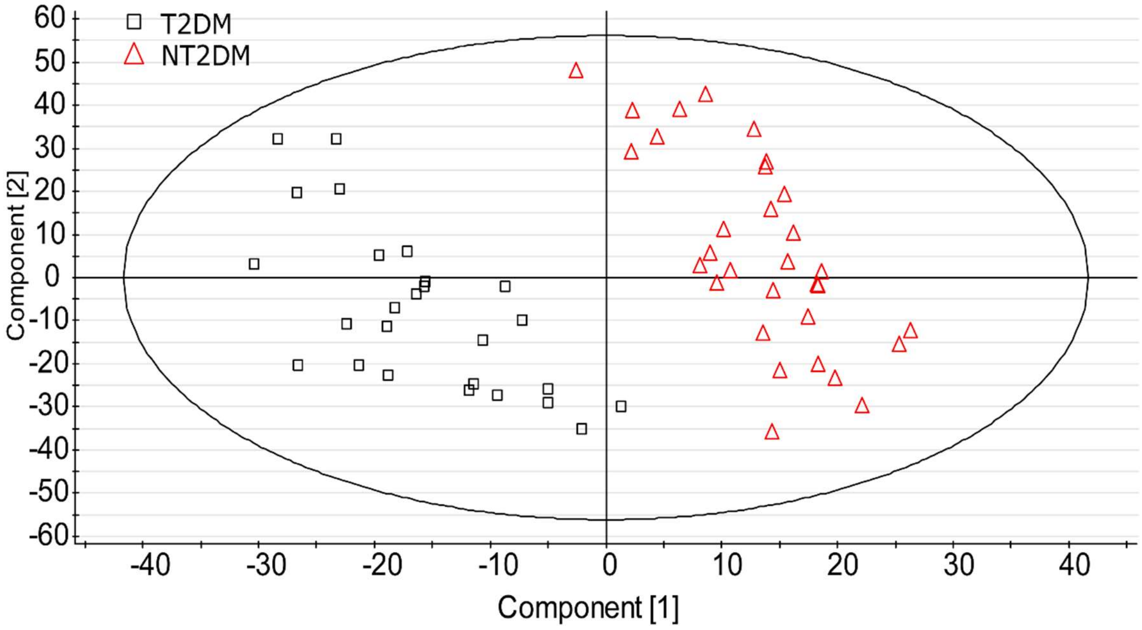

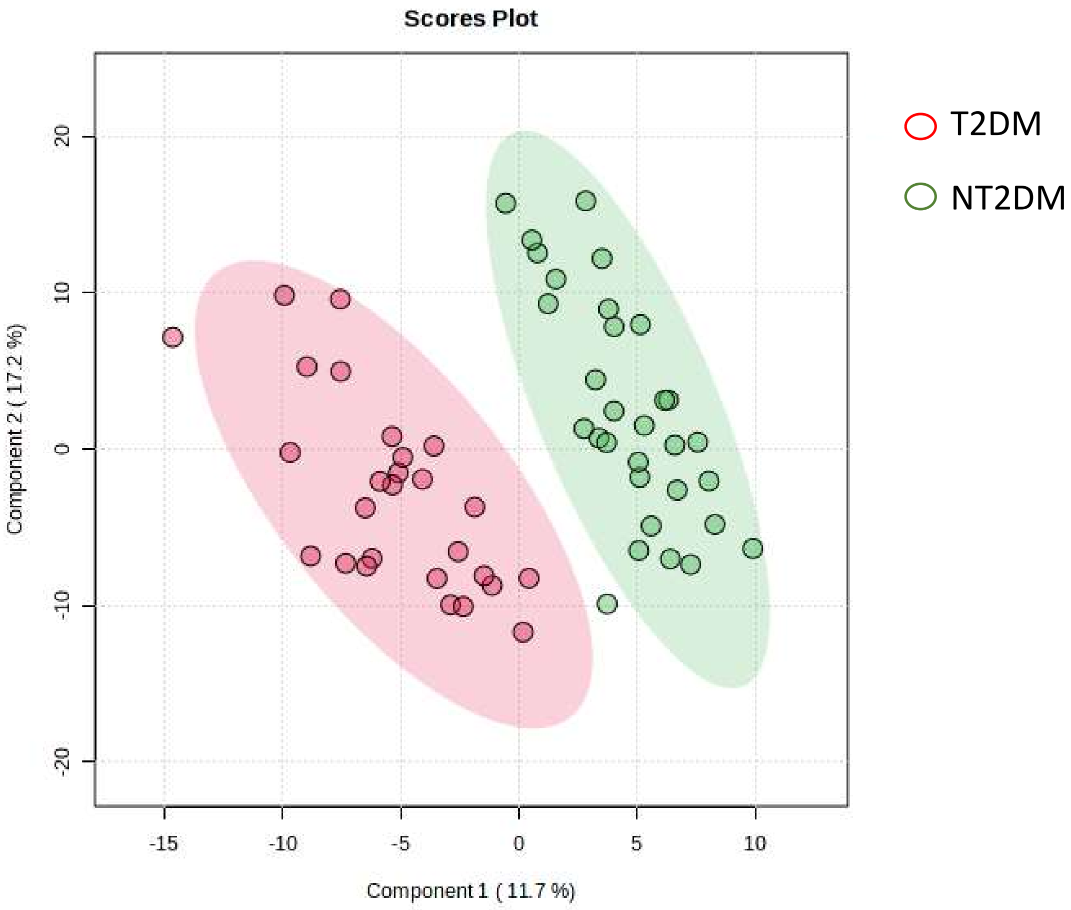

Supplement: Supplementary file 1 [file metabolites-15-00395-s001.zip › Supplementary_Figure_S2.pdf]
