# Supplementary material for: Blood Metabolic Biomarkers of Diabetes Mellitus Type 2 in Aged Adults Determined by a UPLC-MS Metabolomic Approach
Source: Metabolites. 2025 Jun 12;15(6):395. doi: 10.3390/metabo15060395 (PMC12195025; doi:10.3390/metabo15060395)
Supplement: Supplementary file 1 [file metabolites-15-00395-s001.zip › Supplementary_Table_S1.pdf]

Supplementary Table S1. Statistical data of the correlation between "Consumption of sugar-rich foods" and the selected metabolites.

	Consumption of sugar-rich foods			
**Characteristic**	**Overall** N = 59[1]	**No** N = 29[1]	**Yes** N = 30[1]	**p-value**[2]
LPC(22:6)	360.1 (185.6) ; 324.7 [242.7, 443.5]	344.0 (193.6) ; 313.3 [240.7, 403.7]	375.7 (179.5) ; 337.8 [259.3, 454.4]	0.456
LPC(20:4)	1,412.7 (700.2) ; 1,229.8 [1,017.5, 1,896.5]	1,303.9 (686.0) ; 1,229.8 [907.3, 1,450.6]	1,517.8 (709.0) ; 1,237.5 [1,045.9, 2,044.2]	0.411
LPC(14:0)	190.4 (105.5) ; 174.5 [118.1, 228.2]	163.1 (105.1) ; 137.5 [100.3, 204.8]	216.7 (100.7) ; 214.5 [140.7, 253.3]	0.012
Gly-His	60.3 (97.3) ; 15.6 [0.0, 84.3]	49.1 (102.2) ; 0.0 [0.0, 45.6]	71.1 (92.7) ; 42.3 [0.0, 99.5]	0.090
Ganglioside 2	71.8 (45.2) ; 64.3 [42.5, 87.8]	65.4 (51.0) ; 53.8 [29.3, 80.6]	78.0 (38.7) ; 74.4 [56.1, 93.7]	0.075

[1] Mean (SD) ; Median [Q1, Q3]

[2] Wilcoxon rank sum exact test; Wilcoxon rank sum test
